# Supplementary material for: Reengineering the specificity of the highly selective Clostridium botulinum protease via directed evolution
Source: Sci Rep. 2022 Jun 15;12:9956. doi: 10.1038/s41598-022-13617-z (PMC9200782; doi:10.1038/s41598-022-13617-z)
Supplement: Supplementary file 1 — Supplementary Information. [file 41598_2022_13617_MOESM1_ESM.pdf]

Supplementary Material

Reengineering the Specificity of the Highly Selective *Clostridium botulinum* Protease via Directed Evolution

Rebekah P. Dyer[2], Hariny M. Isoda[1], Gabriela S. Salcedo[2], Gaetano Speciale[1], Madison H. Fletcher[1], Linh Q. Le[4], Yi Liu[4], Karen Brami-Cherrier[4], Shiazah Z. Malik[4], Edwin J. Vazquez-Cintron[4], Andrew C. Chu[2], David C. Rupp[4], Birgitte P.S. Jacky[4], Thu T.M. Nguyen[1], Benjamin B. Katz[1], Lance E. Steward[4], Sudipta Majumdar[1], Amy D. Brideau-Andersen[4]*, Gregory A. Weiss[1,2,3]*

Affiliations:
[1]Departments of [1]Chemistry, [2]Molecular Biology and Biochemistry, [3]Pharmaceutical Sciences, University of California, Irvine, 1102 NS-2, Irvine, CA 92697-2025 USA
[4]Allergan Aesthetics, an AbbVie company, 2525 Dupont Drive, Irvine CA 92612 USA
*Correspondence to: Dr. Amy Brideau-Andersen (amy.brideauandersen@abbvie.com) and Dr. Gregory Weiss (gweiss@uci.edu).

Address all correspondence to:
*Dr. Gregory A. Weiss
Departments of Chemistry, Molecular Biology and Biochemistry, and Pharmaceutical Sciences
University of California, Irvine
1102 Natural Sciences 2
Irvine, CA 92697-2025
USA
Phone: 1 (949) 824-5566
Fax: 1 (949) 824-8571

*Dr. Amy D. Brideau-Andersen
Allergan Aesthetics, an AbbVie company
2525 Dupont Drive
Irvine, CA 92612
USA

Table of Contents

Figure S1. .... S2
Figure S2. .... S2
Figure S3. .... S3
Figure S4. .... S4
Figure S5. .... S5
Figure S6. .... S6
Figure S7. .... S6
Figure S8. .... S6-S7
Figure S9. .... S7
Figure S10. .... S8
Figure S11. .... S8
Figure S12. .... S9-S10
Figure S13. .... S10

Figure S14. ....S11

Supplemental Figures

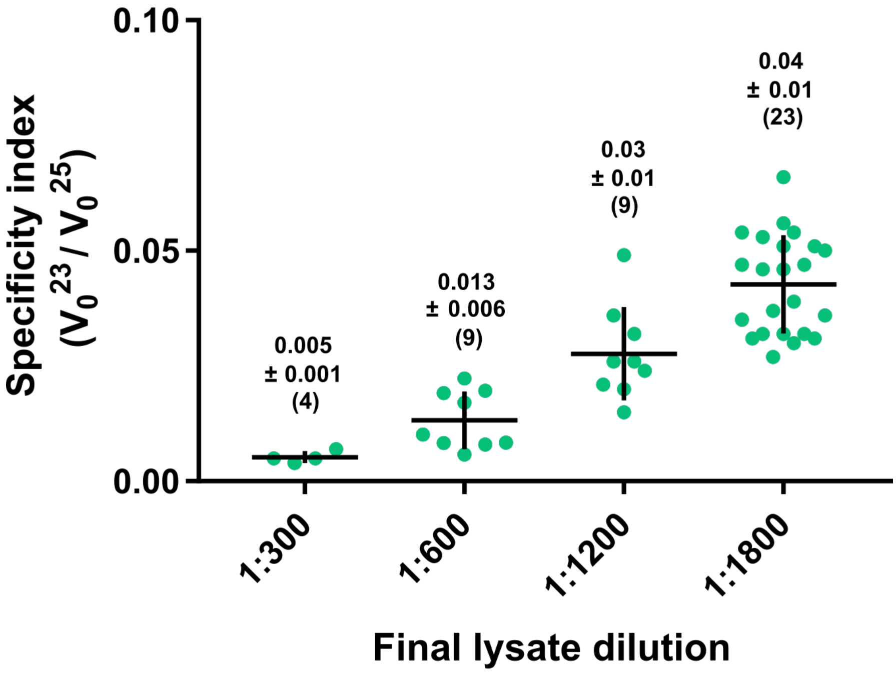

Figure S1. The substrate specificity of the qmLC/A variant. As shown by the specificity index (V_0^{23}/V_0^{25}) at 2 µM substrate, qmLC/A in cell lysate is highly specific for SNAP25. However, diluting the cell lysate increases the SNAP23 specificity of qmLC/A. Each point represents a different technical or biological replicate at the indicated final lysate dilution. The average value and standard deviation are shown as numbers above each dilution with the total number of data points in parentheses. Horizontal lines indicate average value and vertical lines indicate one standard deviation. The four dilutions yielded significantly different specificity indices as analyzed by an unpaired t-test in GraphPad Prism (p = 0.001).

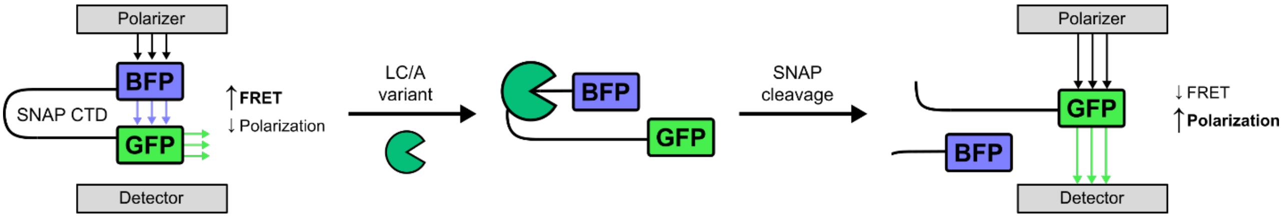

Figure S2. Depolarization After Resonance Energy Transfer (DARET) assay. Cartoon representing the DARET assay for SNAP cleavage by LC/A variants. CTD, C-terminal domain, corresponds to residues 137 to 211 of SNAP25 or residues 134 to 206 of SNAP23.

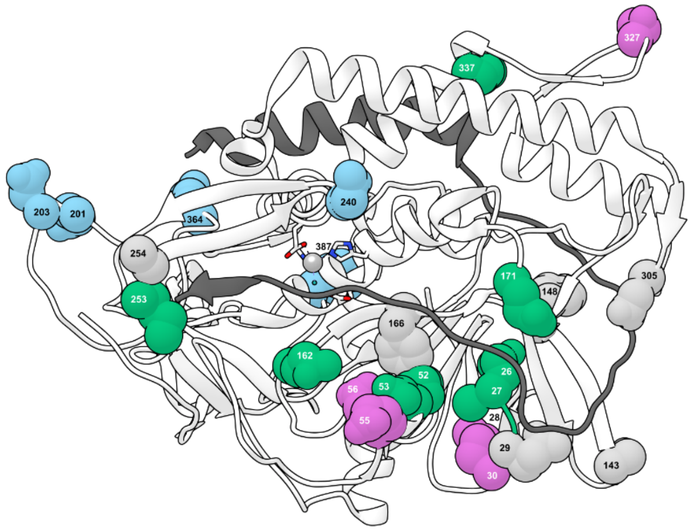

Stochastic (epPCR)
E201, D203, N240, K364, Y387
Previous structural modeling*
Q29, S143, Y148, F166, A254, D305
Structural analysis
N26, A27, G28, T52, N53, Q162, E171, M253, K337
B-factor analysis
M30, E55, E56, T327

Figure S3. Residues targeted and techniques used for directed evolution. Residues highlighted in blue were identified via stochastic methods (error-prone PCR, epPCR). Gray residues were mutated with the NDT and VHG codons (all AAs substituted with the exception of Trp). Sites directing specificity in teal were chosen through structural analysis of the SNAP25-LC/A complex (1XTG, shown here), and were mutated with the NDT codon (12 AAs substituted: Cys, Asp, Phe, Gly, His, Ile, Leu, Asn, Arg, Ser, Val, Tyr). Purple residues were chosen based upon B-factor analysis, and were mutated with the NDT codon. Position 240 was substituted with all 20 AAs using Tang's mutagenesis method. Asterisk (*) indicates the residues reported by Binz et al. (*21*).

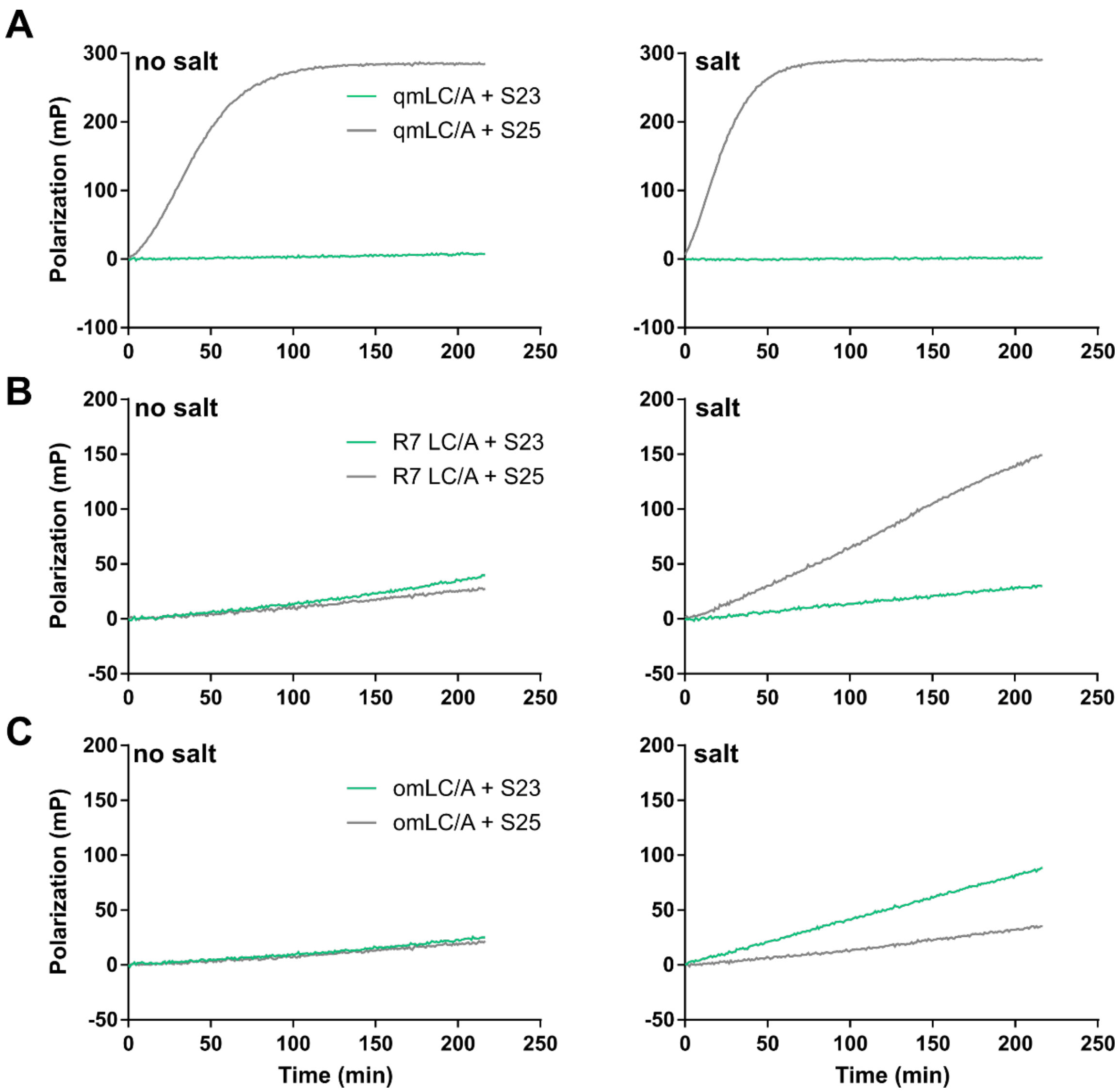

Figure S4. Salt sensitivity of LC/A selectants. In Round 8 of directed evolution, diluted lysates (1:1800) containing qmLC/A, omLC/A, or the most SNAP23 specific variant from Round 7 (R7) were tested for SNAP cleavage in "salt-free" (50 mM HEPES pH 7.4, 0.05% Tween) or salt (50 mM KH_2PO_4 pH 7.4) buffered conditions. SNAP23 and SNAP25 are indicated in green and gray, respectively. (**A**) The qmLC/A variant is highly specific for SNAP25 in both no salt and salt conditions. (**B**) The R7 LC/A variant is specific for SNAP23 in no salt conditions but becomes specific for SNAP25 in salt. (**C**) The omLC/A variant exhibits little SNAP23 or SNAP25 specificity in no salt conditions, but is specific for SNAP23 in salt conditions. Each line depicts the mean of three technical replicates after subtracting a no-enzyme blank. The dotted lines and the shaded area depict the standard deviation around the mean; however, the standard deviation for each condition tested is too small to be visible on the graphs.

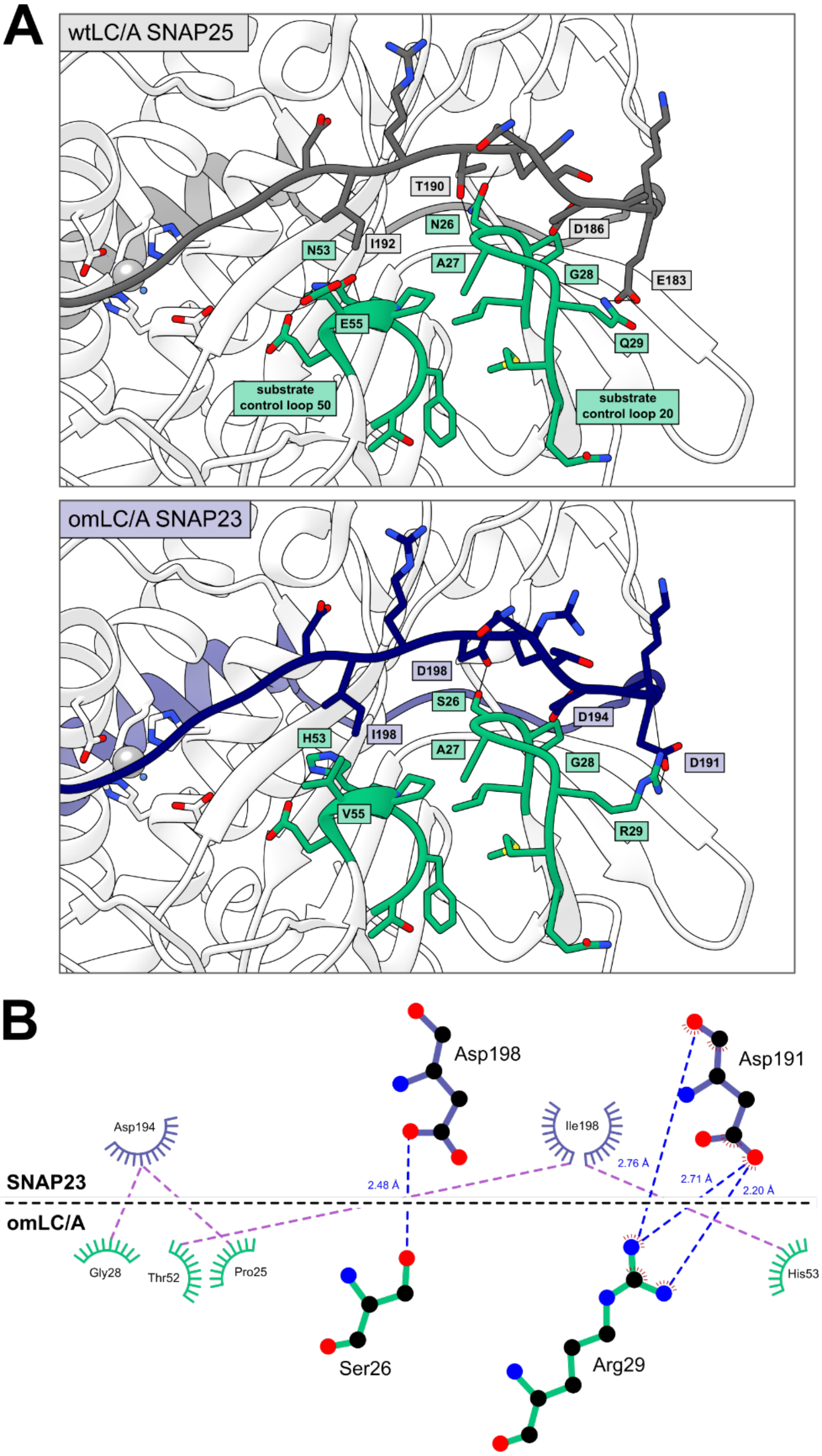

Figure S5. Substrate control loops in LC/A. (**A**) Residues occupying substrate control loops 20 and 50 are highlighted for wtLC/A (top) and omLC/A (bottom, computational modeling). (**B**) Putative modeled protein-protein interactions between SNAP23 sidechains (navy) and omLC/A substrate control loop residues (teal) (LigPlot^{+} software). Hydrophobic interactions are depicted in purple, and putative polar contacts are shown in blue.

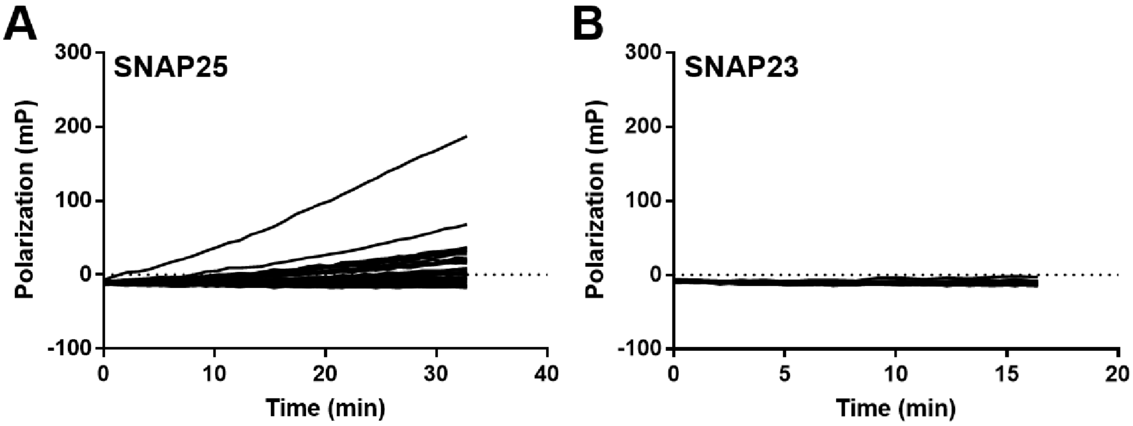

Figure S6. SNAP cleavage activity of K337NDT clones. In Round 6 of directed evolution, 34 variants from the alpha exosite K337NDT LC/A library were tested for (**A**) SNAP25 or (**B**) SNAP23 cleavage in the DARET assay. Each line represents the diluted lysate (1:1800) of a single well of a 96 deep-well plate.

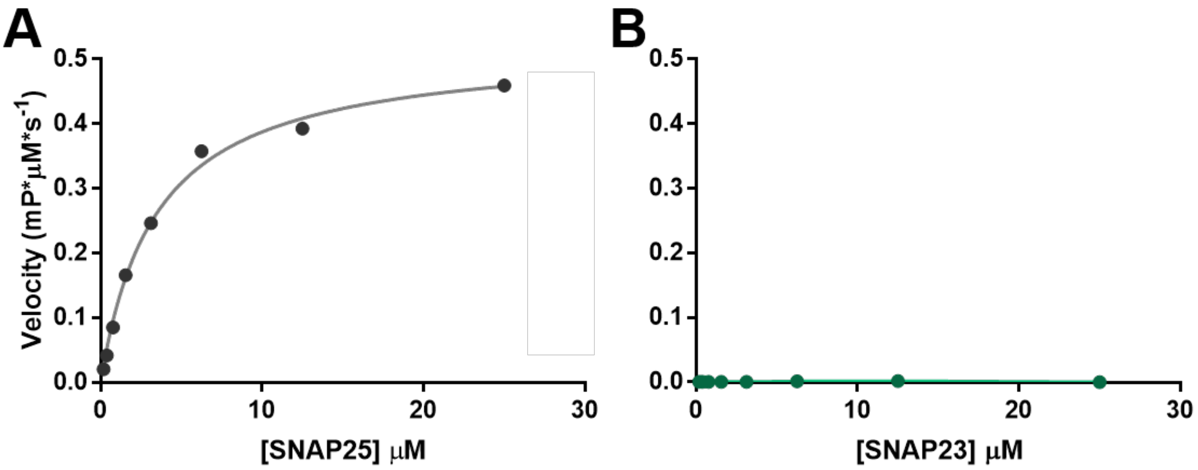

Figure S7. Kinetic characterization of wtLC/A. Rates of (**A**) SNAP25 or (**B**) SNAP23 cleavage by batch-expressed, purified wtLC/A at the indicated DARET substrate concentrations (n=3). There is no detectable SNAP23 cleavage by wtLC/A in this assay.

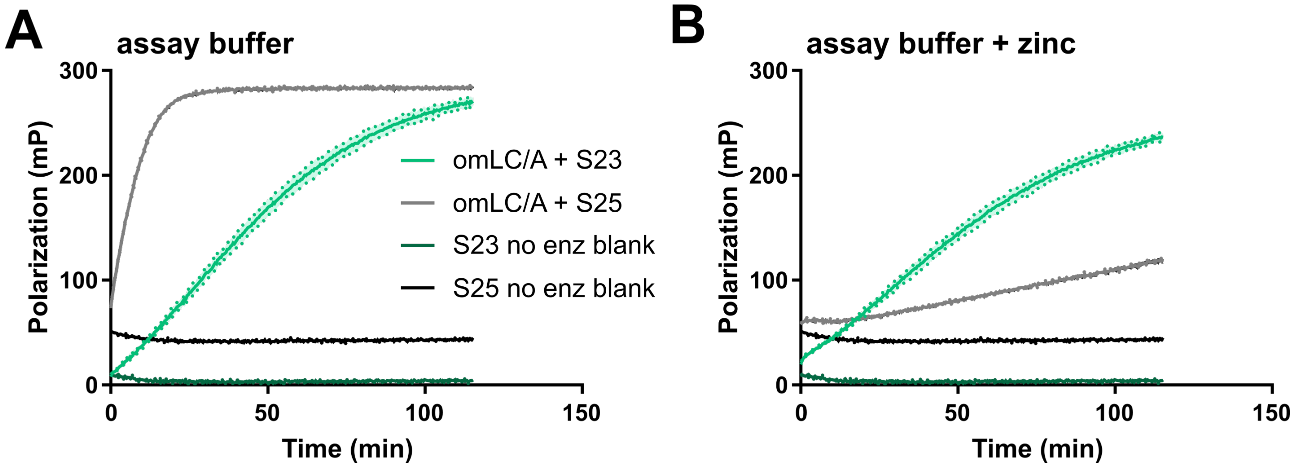

Figure S8. Zinc dependence of omLC/A substrate specificity. (**A**) Batch-expressed, IMAC-purified fractions of omLC/A were dialyzed into either Assay Buffer (50 mM HEPES pH 7.4) or (**B**)

Assay Buffer plus zinc (50 mM HEPES, 2 µM $ZnCl_2$ pH 7.4) for 20 h at 4 °C before characterizing SNAP cleavage by the DARET assay (no enz blank = no enzyme blank negative control). Typically, zinc is not supplemented during the purification of wtLC/A. Each solid line depicts the mean of three technical replicates (50 nM enzyme, 2 µM substrate). The dotted lines and the shaded area depict the standard deviation around the mean.

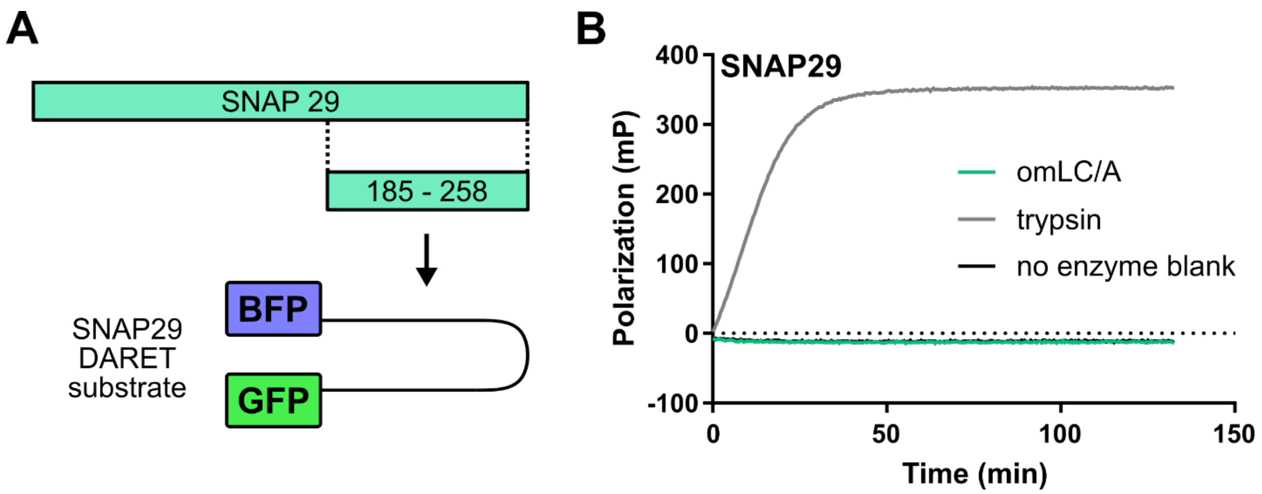

Figure S9. Cleavage of related SNARE SNAP29 by omLC/A. (**A**) Design of SNAP29 DARET substrate for fluorescence polarization assays of proteolytic cleavage, featuring the 73 C-terminal residues of SNAP29. (**B**) The omLC/A variant exhibits no SNAP29 cleavage in the DARET assay. SNAP29 is the closest related SNAP family member to SNAP25 and SNAP23. Trypsin provides a positive control for SNAP29 cleavage**.** Each solid line depicts the mean of three technical replicates (50 nM enzyme, 300 nM substrate). The dotted lines and the shaded area depict the standard deviation around the mean; however, the standard deviation for each condition tested is too small to be visible on the graph.

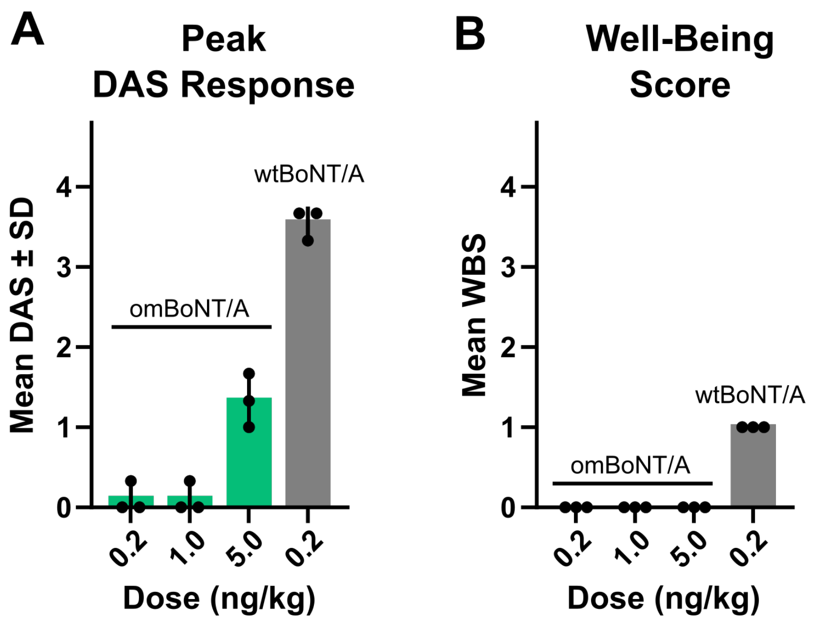

Figure S10. In vivo evaluation of neuromuscular paralysis of omBoNT/A compared to wtBoNT/A. (**A**) Neuromuscular paralysis measured by peak DAS effect in mice (n=3/dose; N=3) injected with omBoNT/A (0.2, 1, or 5 ng/kg) or wtBoNT/A (0.2 ng/kg). The paralytic response is mediated by cleavage of SNAP25. (**B**) Mice injected with omBoNT/A (n=3/dose; N=3) had no changes in the well-being score (WBS) over four days. These results confirm reduced SNAP25 activity *in vivo* and demonstrate that omBoNT/A does not result in increased overt toxicity.

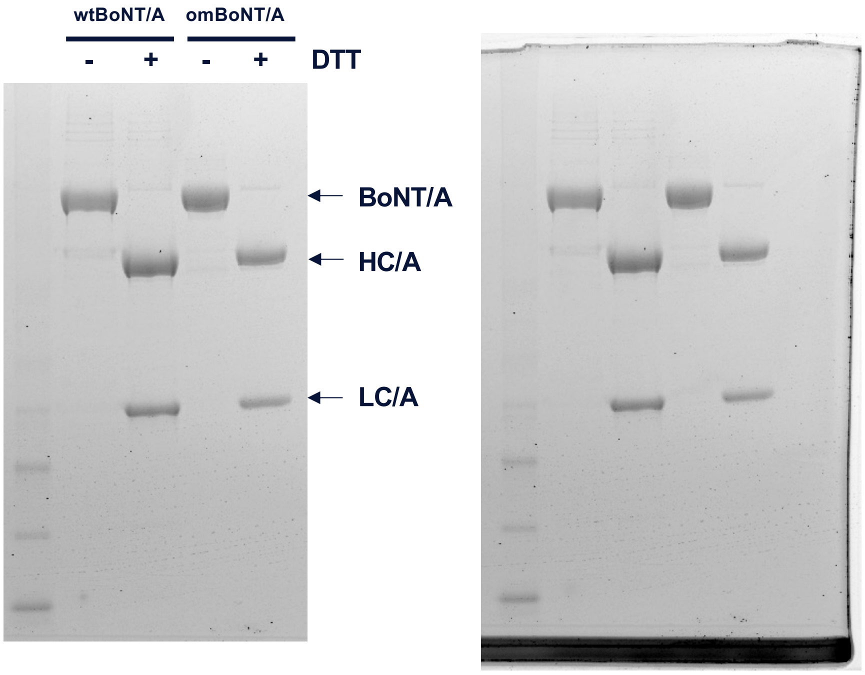

Figure S11. SDS PAGE comparing wtBoNT/A and omBoNT/A. The full-size, uncropped gel is shown on the right.

A

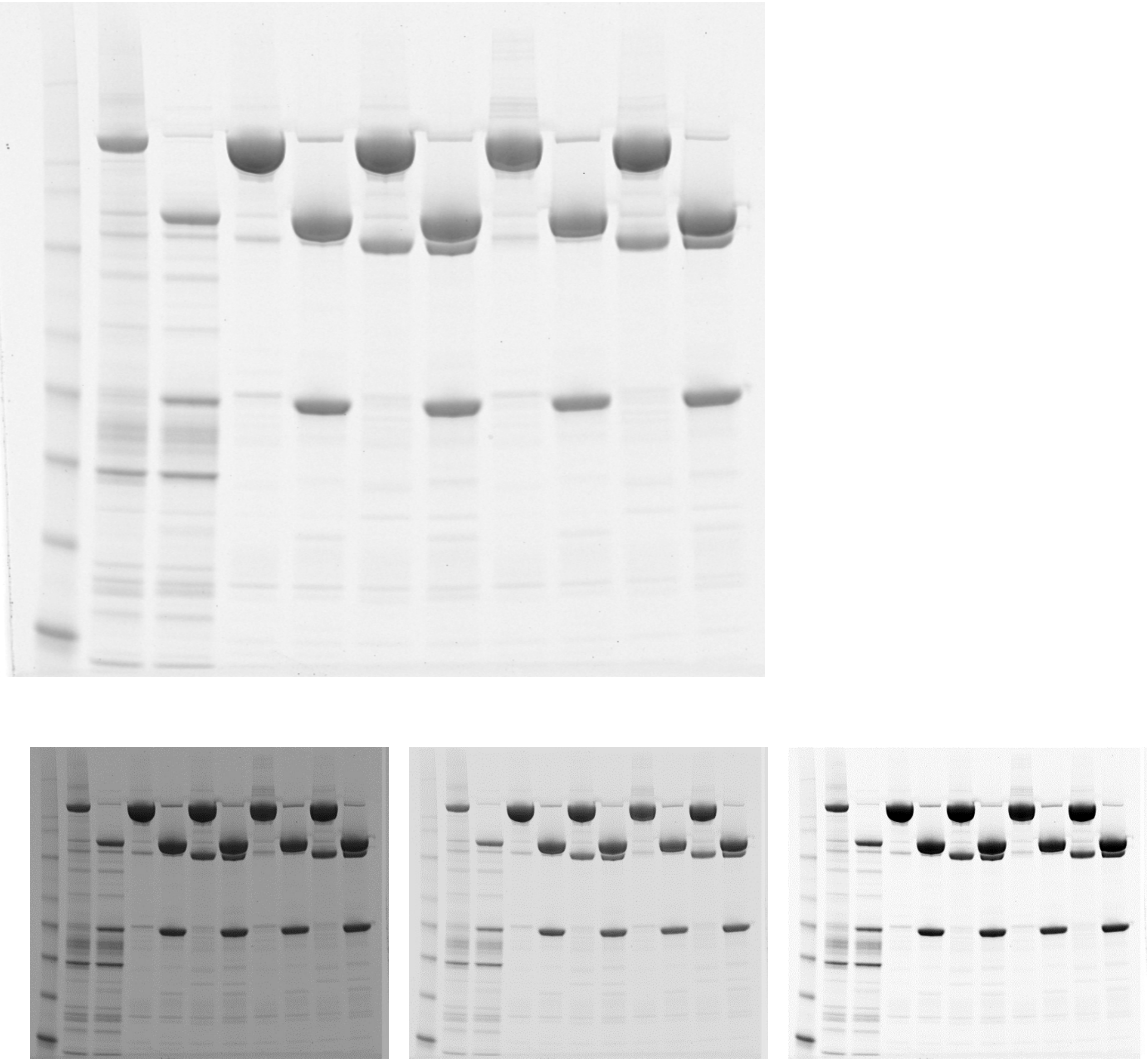

B

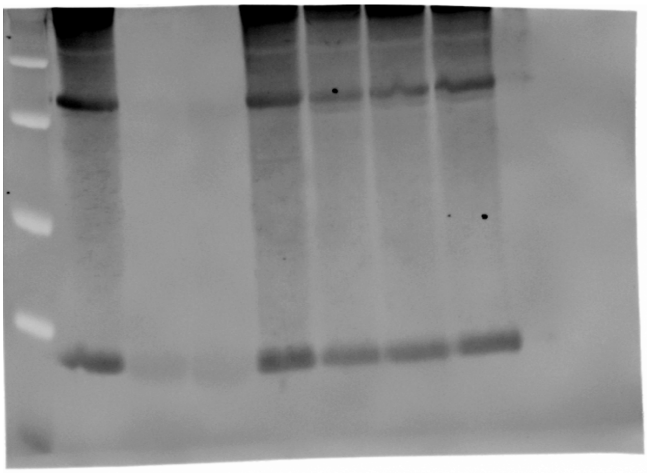

C

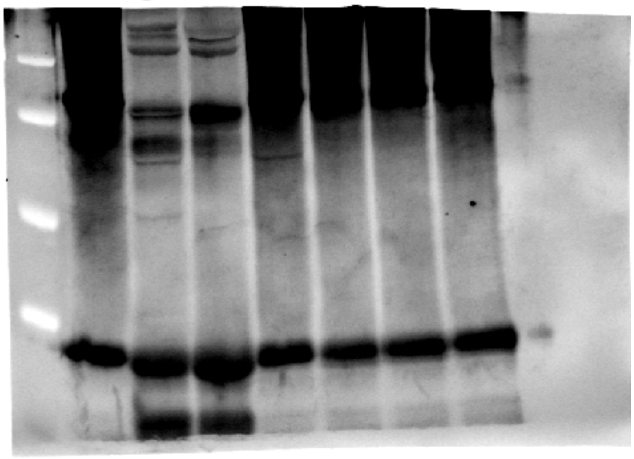

Figure S12. Full-length gels and blots (previous page). (**A**) The full-length gel as shown in **Fig. 3D.** Additional exposures with different contrasts are shown below. (**B**) The full-length gel as shown in **Fig. 3E** (**C**). The same blot visualized in panel **B** was then washed extensively with TBST and re-probed with anti-SNAP23 antibody (Abcam ab4114). This second blot demonstrates that the samples had similar levels of recombinant SNAP23 protein. The loss of SNAP23 observed in the first blot (panel **B**) is likely due to cleavage of the protein and consequent loss of the epitope to the antibody used.

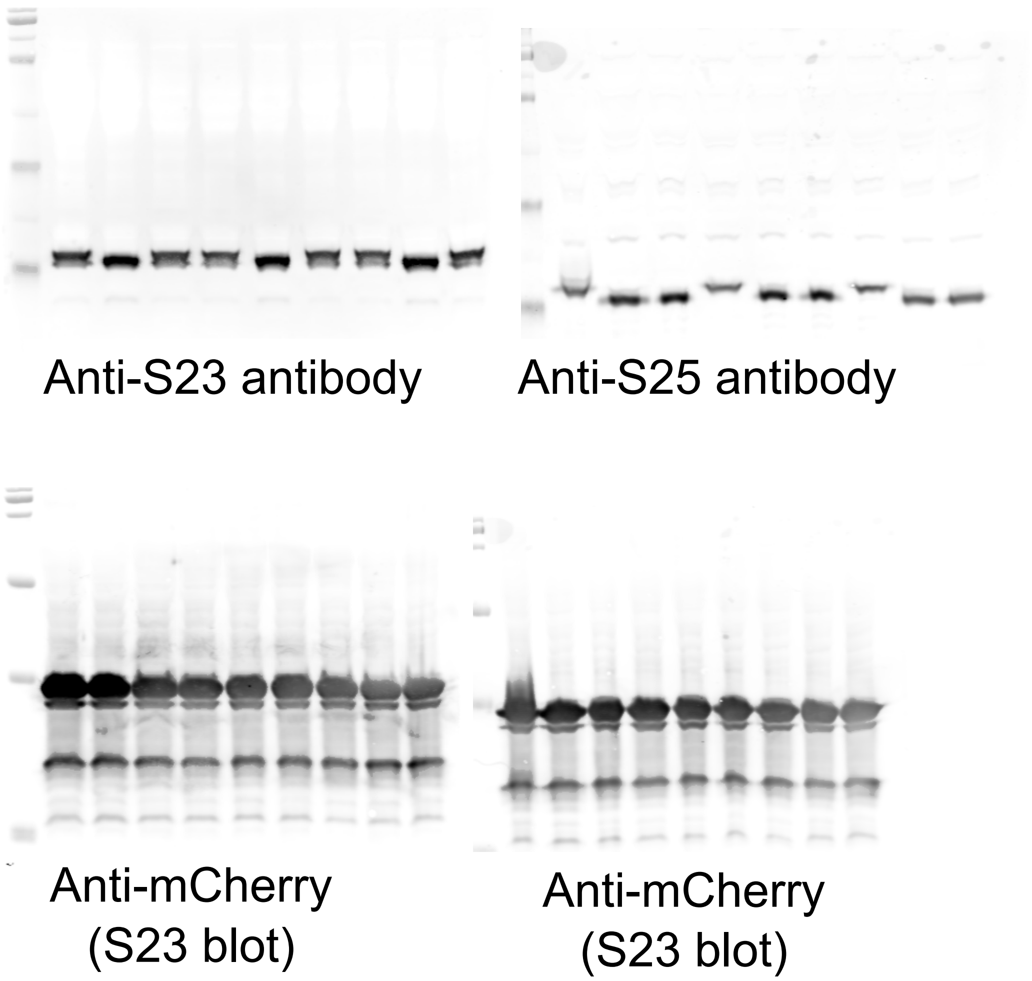

Figure S13. Full-length blots. The full-length blots as shown in **Fig. 3F**.

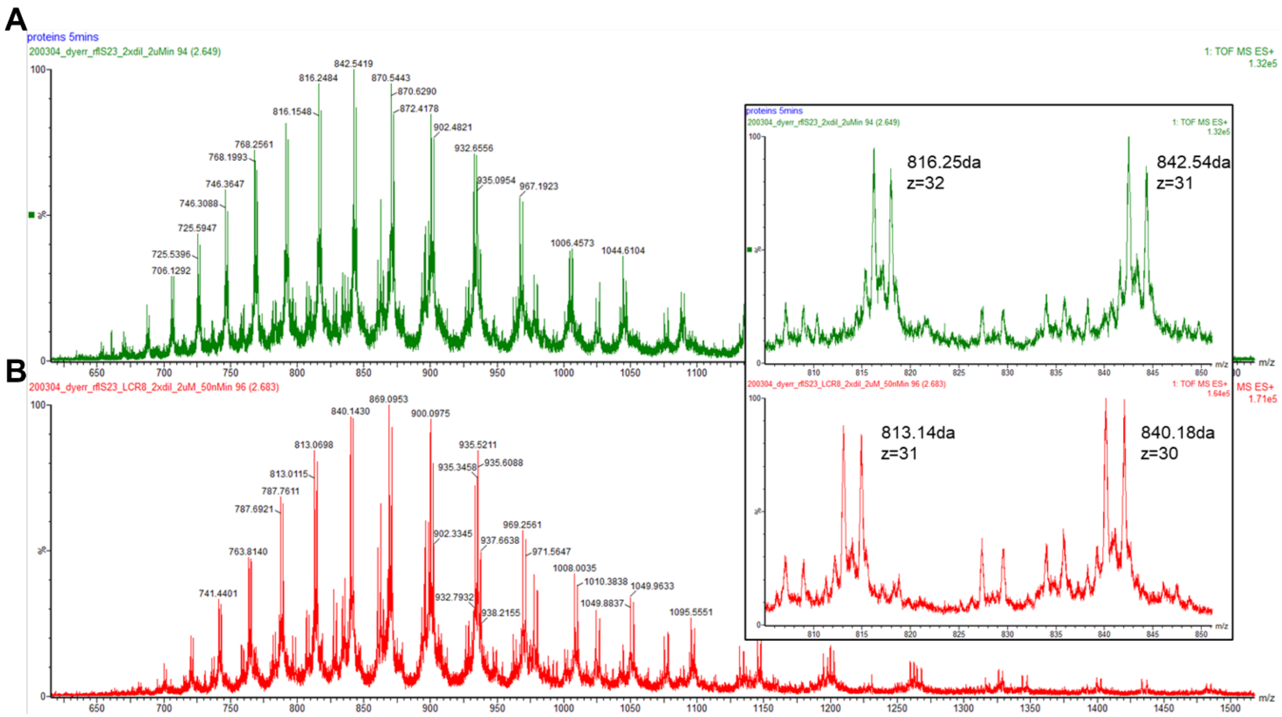

Figure S14. Chromatography and mass spectrometry of full-length SNAP23 before and after cleavage by omLC/A. Un-deconvoluted mass spectra of (**A**) intact and (**B**) omLCA-cleaved full-length, recombinantly-expressed SNAP23. The x-axis represents mass per charge (m/z) and the y axis indicates percent of total ions (%). The inset graph for each spectrum shows two charge states (z) of the protein charge ladder for each sample.
